# Conformational preludes to the latency transition in PAI-1 as determined by atomistic computer simulations and hydrogen/deuterium-exchange mass spectrometry

**DOI:** 10.1038/s41598-017-06290-0

**Published:** 2017-07-26

**Authors:** Michael Petersen, Jeppe B. Madsen, Thomas J. D. Jørgensen, Morten B. Trelle

**Affiliations:** 1 Department of Physics, Chemistry and Pharmacy, University of Southern Denmark, Campusvej 55, 5230 Odense, M Denmark; 2 Department of Biochemistry and Molecular Biology, University of Southern Denmark, Campusvej 55, 5230 Odense, M Denmark

## Abstract

Both function and dysfunction of serine protease inhibitors (serpins) involve massive conformational change in their tertiary structure but the dynamics facilitating these events remain poorly understood. We have studied the dynamic preludes to conformational change in the serpin plasminogen activator inhibitor 1 (PAI-1). We report the first multi-microsecond atomistic molecular dynamics simulations of PAI-1 and compare the data with experimental hydrogen/deuterium-exchange data (HDXMS). The simulations reveal notable conformational flexibility of helices D, E and F and major fluctuations are observed in the W86-loop which occasionally leads to progressive detachment of β-strand 2 A from β-strand 3 A. An interesting correlation between C_α_-RMSD values from simulations and experimental HDXMS data is observed. Helices D, E and F are known to be important for the overall stability of active PAI-1 as ligand binding in this region can accelerate or decelerate the conformational inactivation. Plasticity in this region may thus be mechanistically linked to the conformational change, possibly through facilitation of further unfolding of the hydrophobic core, as previously reported. This study provides a promising example of how computer simulations can help tether out mechanisms of serpin function and dysfunction at a spatial and temporal resolution that is far beyond the reach of any experiment.

## Introduction

Serine protease inhibitors (Serpins) are a fascinating group of proteins due to their profound conformational flexibility. Most serpins (but not all) are indeed inhibitors of serine proteases and change conformation when they react with their target protease^1^. This essential conformational plasticity also renders many serpins prone to pathological changes in their structure, such as conversion to an inactive “latent” form or to polymerization. The most well-known serpin-related disease is α_1_-antitrypsin deficiency, affecting 1 in 1700 persons in the northwestern European population, but many other exists, collectively referred to as “serpinopathies”^2^. Our understanding of the intrinsic dynamics facilitating conformational change in serpins is incomplete and efforts to intervene pharmaceutically have, as a result, been largely futile.

**Figure 1:**
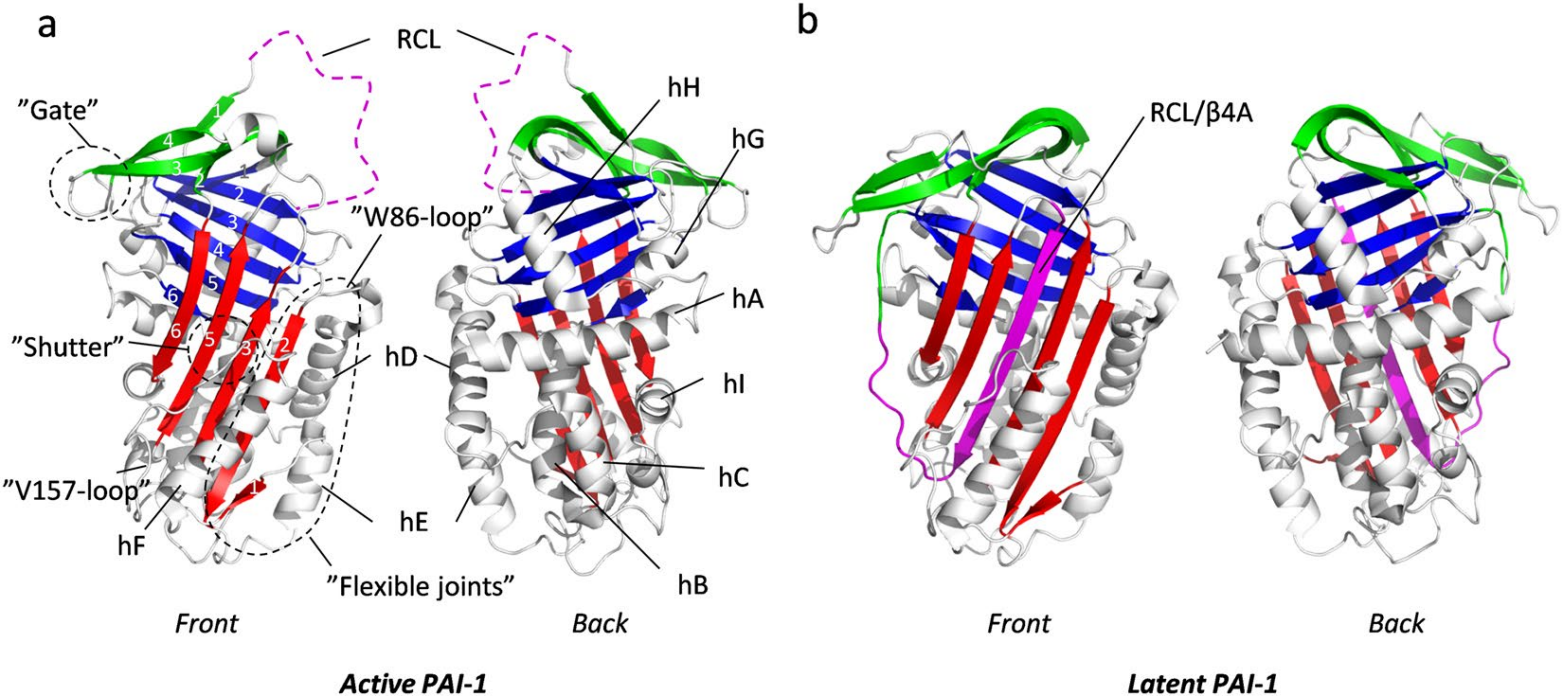
Structure of active and latent PAI-1. (**a**) crystal structure of the W175F mutant of PAI-1 in the active conformation (PDB 3Q02). β-sheet A, B and C are shown in red, blue and green, respectively. RCL residues missing in the PDB 3Q02 are sketched as a purple broken line. Other secondary structures and named regions are indicated in the figure. (**b**) Structure of wild-type PAI-1 in the latent conformation (PDB 1DVN). Color coding is the same as in (**a**). In this conformation the RCL (purple) is inserted as an extra β-strand in the middle of β-sheet A.

Serpins natively fold to an active conformation containing 3 β-sheets, named A, B and C, as well as 8–9 α-helices named hA to hI (Fig. 1a). Protease inhibition is instigated by protease cleavage of a surface-exposed reactive center loop (RCL) in the serpin and covalent attachment of the protease to the N-terminal half of the RCL. This event is followed by a massive 70 Å translocation of the protease to the opposite pole of the serpin and insertion of the protease-linked part of the RCL into the central β-sheet A of the serpin. This translocation distorts the active site of the entrapped protease and inhibits its ability to release itself from the inhibitory complex with the serpin^1^.

Many serpins are prone to inactivating conformational changes, often as a result of destabilizing mutations. One inactivation mechanism is conversion to the “latent“ form, in which the RCL is inserted into β-sheet A without prior cleavage by the protease (Fig. 1b). Latency transition occurs spontaneously in certain disease causing serpin mutants^3, 4^ but also in wild-type plasminogen activator inhibitor 1 (PAI-1)^5^. While latency transition in PAI-1 is likely to play a key role in regulating PAI-1 activity locally, and thus is not pathological *per se*, we study PAI-1 as a prototypic example of this conformational change in serpins. Under physiological conditions, the half-life of PAI-1’s latency transition is 1–2 h^6–10^. It has been generally assumed that latency transition may occur without significant unfolding of the serpin fold, but through a step-wise opening of β-sheet A and insertion of single RCL residues one at a time in a “zipper”-like fashion^1, 11–18^. However, using hydrogen/deuterium-exchange monitored by mass spectrometry (HDXMS) we previously discovered unanticipated transient structural unfolding in the native ensemble of PAI-1 at physiological temperature, salt and pH^19^. Transient unfolding was observed on the minute timescale around β-strand 6B and the top of helix B as well as β-strand 5A, in and around the area where the RCL is being inserted. Furthermore, an area around the lower part of helix B and C was observed to transiently unfold at faster timescales (seconds). The details of how and where these unfolding events are initiated and how they are mechanistically linked to the process of latency transition in PAI-1 remains to be elucidated. In this respect it is interesting to note that limited proteolysis^20^ and HDXMS experiments^21–23^ have previously indicated high conformational flexibility in the area around helices D, E and F, which is often referred to as the “flexible joints” region (Fig. 1a). Furthermore, ligands which affect the rate of latency transition are known to bind this region of the protein^21–25^, indicating that the flexible joint region functions as a “control surface” for PAI-1 stability. The mechanistic details of these effects are poorly understood.

Previous HDXMS experiments on PAI-1 were conducted at 25 °C or 37 °C^19, 21–23^. At these temperatures a number of peptides from the flexible joints region reach close to full deuteration within the first labeling time point. The dynamics of this region, at these temperatures, are thus too fast to be efficiently resolved by HDXMS. To provide novel high-resolution spatial and temporal insight to the fastest local dynamics of PAI-1, including the flexible joints region, we therefore decided to perform molecular dynamics simulations in atomistic detail and explicit solvent. Molecular dynamics simulations represent a rapidly evolving methodology to achieve basic insight to the structural fluctuations in protein molecules. While a number of serpins have previously been subjected to “free” molecular dynamics simulations in atomistic detail^26–31^, only simulations that artificially force the active to latent transition have to our knowledge previously been performed on PAI-1^18, 32^. We have conducted microsecond-long molecular dynamics simulations on wild-type PAI-1 in both the active and latent conformations. To attempt a better experimental sampling of the faster dynamics in PAI-1 with HDXMS to complement the simulations, we have probed the backbone dynamics by HDXMS at 5 °C in solution. Our results show notable flexibility in the area around helices D, E and F at the simulated timescales and provide new high-resolution insight to the intrinsic dynamics of PAI-1.

## Results

### Molecular dynamics simulations of active and latent PAI-1

Atomistic molecular dynamics simulations of PAI-1 in the active and latent conformations were conducted in explicit solvent (water molecules was included in the simulation). Four replicate one-microsecond simulations were performed on active PAI-1 and two on latent PAI-1. Additionally, one simulation of active PAI-1 was extended to two microseconds. Furthermore Accelerated Molecular Dynamics (AMD) simulations were conducted^33^. In AMD simulations, minima (below a certain threshold) in the energy landscape are raised by applying “boosts” to the potential energy function. The effect is lowered energy barriers between conformational states and thus more of the energy landscape is likely to be sampled during the simulation time. One-microsecond simulations with either 1× AMD or 2× AMD boosts to the potential energy function were conducted *(see materials and methods)*.

The overall stability of the simulations was evaluated based on average C_α_-RMSD values calculated relative to the first frame of the simulations (Figure S1) and on average structures obtained from the simulations (Figure S2) as described in *Supporting Results*. An increase in the C_α_-RMSD value of active PAI-1 is observed as a function of simulation time (Figure S1a), indicating a rather slow and incomplete equilibration to the simulation conditions, whereas only a very modest increase is observed for latent PAI-1 (Figure S1d). The contributions to the increase of the C_α_-RMSD for the active PAI-1 comes mainly from the RCL and to a smaller extend residues 50–160 which include the W86 and V157 loops (see below). Extension of the simulation time to 2 microseconds for replicate 1 did not indicate a continued increase in the average C_α_-RMSD, suggesting that a local energy minimum is reached in this simulation (Figure S1g). While the simulation of active PAI-1 with 1× AMD boost displayed a slightly increased but stable C_α_-RMSD value in the last 500 ns of the simulation, the simulation with 2×AMD boost was clearly unstable and therefore not analyzed further. The average C_α_-RMSD value is almost 60% higher in the active (3.20 ± 0.52 Å) compared to the latent (2.03 ± 0.06 Å) PAI-1 simulations (Figure S1j). Omission of RCL residues from the calculation substantially reduced the average C_α_-RMSD value obtained from simulations on active PAI-1, arguing for considerable RCL fluctuations during simulations (Figure S1).

### Simulations reveal localized conformational flexibility of active PAI-1 in solution

**Figure 2:**
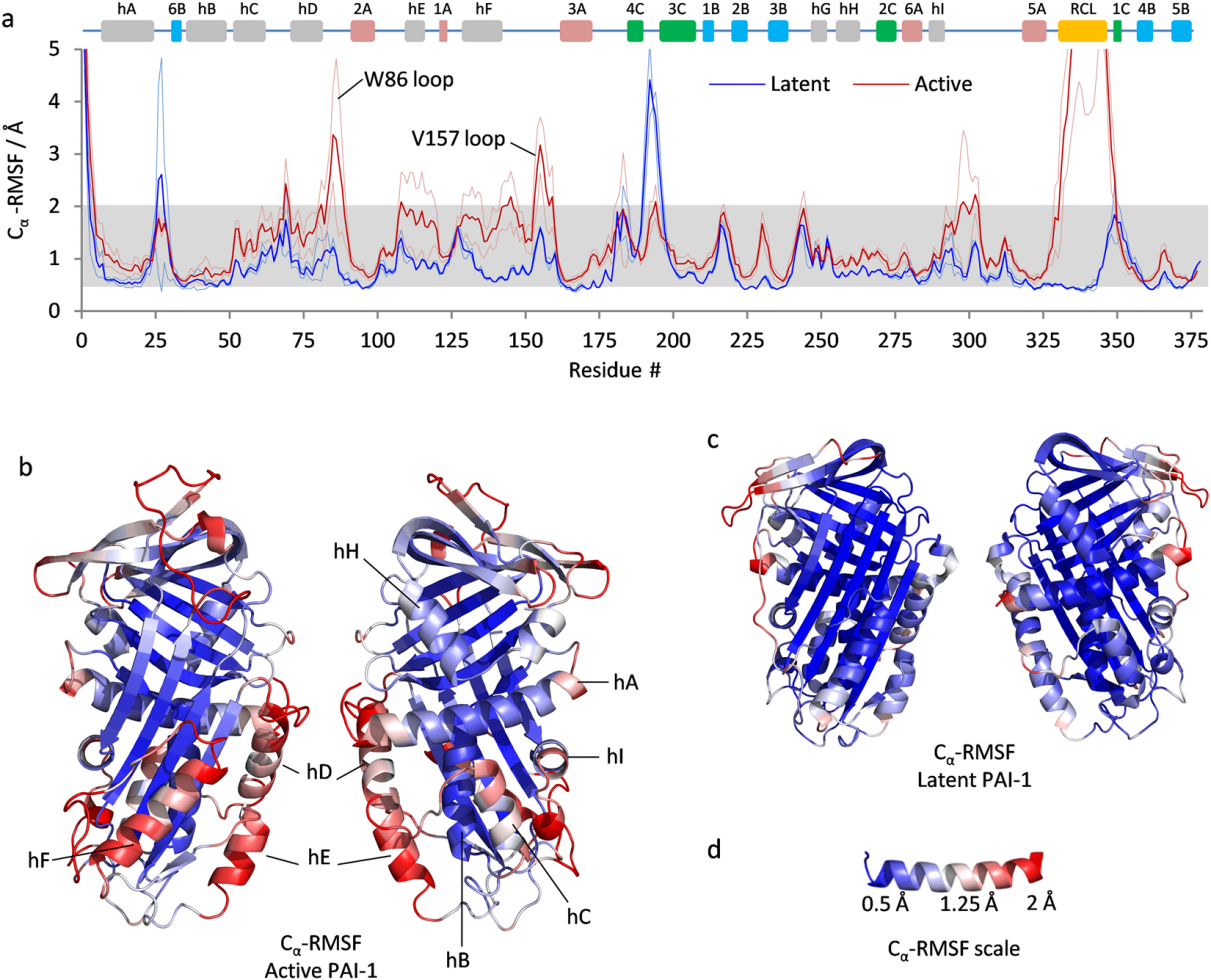
Localized dynamics in active and latent PAI-1. (**a**) The average C_α_-RMSF values from the four replicate one-microsecond simulations on active PAI-1 and the two one-microsecond simulations on latent PAI-1 are indicated as bright red and blue lines, respectively. The average C_α_-RMSF values +/− one standard deviation are shown for active and latent PAI-1 as pale red and pale blue lines, respectively. Secondary structural elements are depicted above the plot for reference. The RCL is colored orange, α-helices are colored grey, β-strands from β-sheet A, B and C are colored red, blue and green, respectively. The average C_α_-RMSF values from the simulation on active PAI-1 and the simulation on latent PAI-1 are indicated on the structure of (**b**) active and (**c**) latent PAI-1 according to the color scale shown in (**d**). The range of this color scale is indicated by the grey area in (**a**).

To investigate the local dynamics in PAI-1 we calculated the residue-specific C_α_-RMSF values relative to the average structure. Values averaged over all replicate simulations on active and latent PAI-1 are plotted in Fig. 2a and values from all individual simulations are plotted in Figure S3. These values are also visualized as a heat map on the structure of active and latent PAI-1 in Fig. 2b,c. The structural elements with the lowest average C_α_-RMSF values in active PAI-1 are β-sheets A and B, hB and the middle part of hA. These structural elements make up most of the solvent-inaccessible hydrophobic core in active PAI-1 and are thus expected to display low amplitude atomic fluctuations. The highest C_α_-RMSF values is observed in the RCL, the W86-loop, between helix D and β-strand 2A, the V157-loop between helix F and β-strand 3A and the loop region following helix I (Fig. 2a). This is accurately reflected in the variation observed in these loops in the average structures of replicate simulations (Figure S2). Interestingly, particularly high C_α_-RMSF values are also observed in secondary structural elements, such as helix C, D, E, F and the termini of helix A (Fig. 2a,b). These helices all contain solvent-exposed surfaces, but helix H as an example also has a solvent-exposed surface, but yet displays rather low C_α_-RMSF values. Solvent exposure alone is thus a poor determinant for flexibility. Taken together, these residue-specific C_α_-RMSF values argues for rather extreme differences in the local fluctuations of active PAI-1 at the microsecond timescale and identifies “hot spots” for conformational flexibility around helix C, D, E, F, the loop following helix I and the RCL.

A general reduction in C_α_-RMSF values is observed in latent PAI-1 relative to active PAI-1 (Fig. 2a,c), with RCL residues experiencing the most dramatic change as they are inserted as the 4^th^ β-strand into β-sheet A. A few regions showed elevated C_α_-RMSF values in the latent simulations compared to the active, including residues 188–200 (β3C-β4C hairpin) and 350–359 (β1C and following loop).

A clear shift towards higher C_α_-RMSF values is observed for most residues in the simulation with 1× AMD boost, but especially residues 50–160 are affected (Figure S3c and S3e). This observation provides further evidence of particular instability in this area of active PAI-1 and directly suggests that input of additional energy into the system results in increased structural dynamics in this region.

### Microsecond simulations on PAI-1 dynamics are in good agreement with Hydrogen/deuterium-exchange experiments

**Figure 3:**
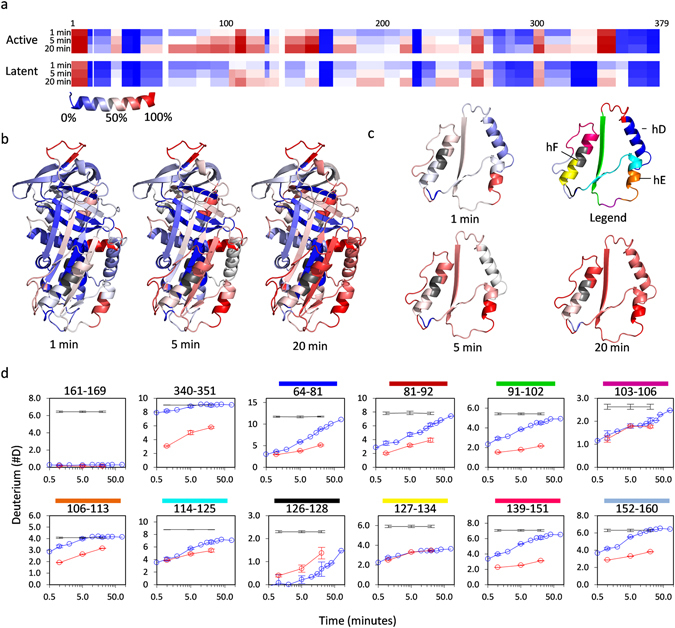
Hydrogen/deuterium-exchange mass spectrometry on active and latent PAI-1 at 5 °C. 40 pmol of active PAI-1 was diluted 100-fold into deuterated PBS pD 7.4 and incubated for 0.5, 1, 2, 5, 10, 15, 20, 30, 40 and 80 minutes at 5 °C prior to proteolytic digestion and analysis by LCMS. Latent PAI-1 was analyzed in a similar manner although only after 1, 5 and 20 minutes incubation. All 1, 5 and 20 minute time points were conducted in triplicate. (**a**) Heat map representing the deuterium content of individual peptides relative to a full deuteration control (see methods) after 1, 5 and 20 minutes incubation. (**b**) The HDXMS heat map of active PAI-1 from (**a**) shown on the active PAI-1 structure (PDB 3Q02) with the W175F substitution and RCL modelled in using Modloop^41, 42^. Grey areas represent sequences not covered by peptide HDX data. Similar representation of the latent PAI-1 HDXMS data is shown in Figure S5. (c) Heat map from (**a**) shown on residues 66-161 only. The upper right depiction is a color map to locate peptides for which deuterium uptake plots are shown in (**d**). (**d**) deuterium uptake plots of the indicated peptides. Active and latent PAI-1 are indicated by the blue and red line, respectively, and the full deuteration level by the black line. Error bars represent the standard deviation from triplicate measurements.

Previous HDXMS experiments on PAI-1 were conducted at 25 °C or 37 °C where a number of peptides reach close to full deuteration within the first labeling time point^19, 21, 22^. To achieve a better kinetic resolution of the isotopic exchange in these peptides and to facilitate a comparison with simulation data reported here, we conducted HDXMS experiments at 5 °C on both active and latent PAI-1 ***(***Figs 3 and S4). Peptides from the hydrophobic core of active PAI-1 acquire essentially no deuterium at 5 °C, as exemplified by peptides 161–169 (Fig. 3d), 34–40 and 372–379 (Figure S4), indicating that the core of active PAI-1 is extremely stable at this temperature. A few regions in active PAI-1 still acquire most of the deuterium prior to the first labeling time point (30 sec), including the RCL (peptide 340–351) and the N-terminal part of Helix A (peptide-16–11) and lower part of helix E (peptide 106–113) (Figs 3 and S4). Although most peptides in helix D and the flexible joint region (residues 61–161) still exchange a number of amide hydrogens for deuterium already within the first 30 seconds, the remaining amide hydrogens exchanges on the minute timescale (Fig. 3d). On the contrary, peptide 127–134 (lower helix F) still contains a number of amide hydrogens after 80 minutes, indicating a segment of relative stability in helix F at 5 °C. As previously observed at 25 °C^22^, most regions of PAI-1 are stabilized upon transition to the latent conformation and as a result incorporates less deuterium at 5 °C (Fig. 3, Figures S4 and S5). Only sub-peptides 126–128 and 267–272 and peptides 185–202, 236–249 and 352–359 showed higher deuterium content in the latent relative to the active conformation of PAI-1.

**Figure 4:**
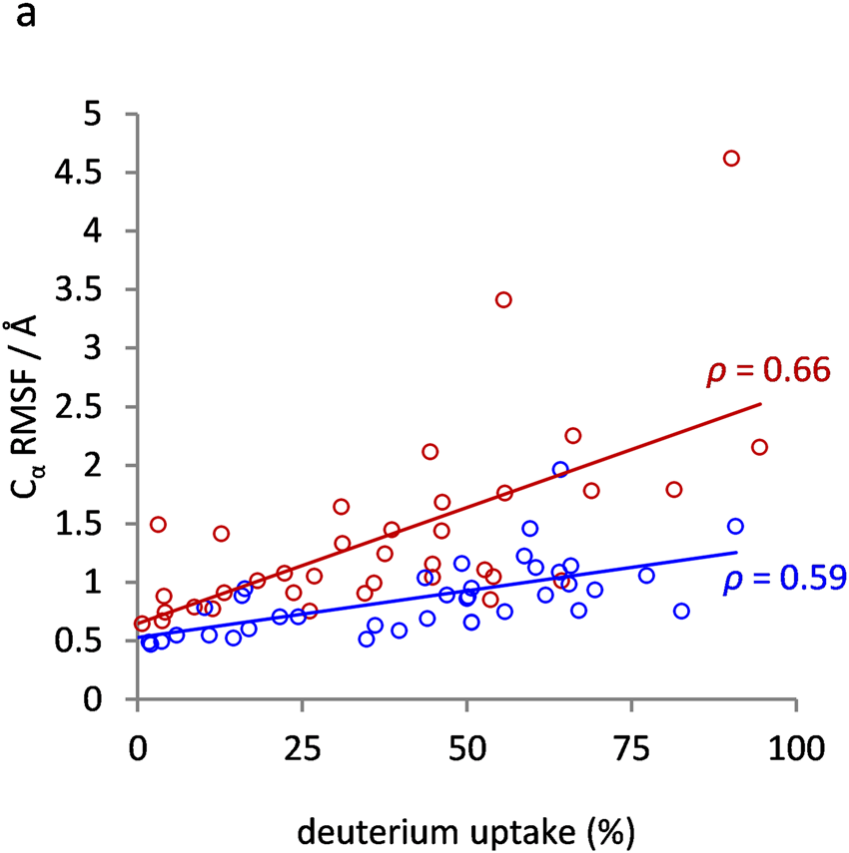
Comparison of simulation C_α_-RMSF values with hydrogen/deuterium-exchange data. Amino acid specific C_α_-RMSF values (from Fig. 2a) averaged for the residues in each of the peptides analyzed in the HDX experiment as well as relative deuterium uptake after 1 minute exposure to D_2_O of active and latent PAI-1 are plotted for active and latent PAI-1 in Figure S6. In the present figure the correlation between C_α_-RMSF values and relative deuterium uptake for active (red) and latent (blue) peptides (taken from Figure S6) is investigated by plotting the two data sets on each axis. Trendlines and Pearson correlation coefficients *ρ* are shown.

As C_α_-RMSF values of molecular dynamics simulations and experimental HDX kinetics of protein backbone amides both relate to the structural flexibility in proteins a correlation between these types of data is expected. The resolution of the C_α_-RMSF data was reduced by averaging C_α_-RMSF values to mimic the peptide-resolution of the HDX data and thereby enable a comparison with the relative deuterium uptake data after 1 minute exposure to D_2_O (Figs 4 and S6). Pearson correlation analysis yielded *ρ*-values of 0.66 and 0.59 for the active and latent PAI-1 data sets respectively. When the differences between active and latent data were compared the *ρ*-value increased to 0.81 (Figure S6). Although the correlation is imperfect, as expected, the fact that some correlation exist indicates that computer simulations of this type can produce meaningful data on the fast fluctuations in serpins.

### Low frequency – large amplitude motions

**Figure 5:**
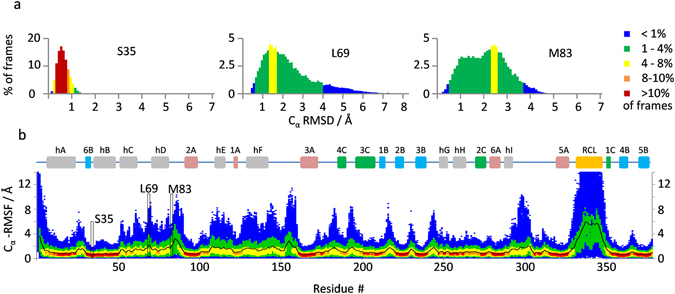
Low frequency – large amplitude motions in active PAI-1. The per-residue, per-frame C_α_-RMSD values relative to the average structure was calculated once every 200 ps for the four simulations on active PAI-1 (total of 20000 frames). (**a**) For each residue, the frames used for calculations were grouped in C_α_-RMSD value bins of 0.1 Å size and the %-fraction of frames in each bin was plotted as histograms according to the indicated color scale. (**b**) Data for S37, L69 and M83 in active PAI-1 are shown as examples. The histograms were then collapsed to a 1-dimensional representation and rotated 90° counter clockwise to facilitate the construction of a density plot of the binned C_α_-RMSD data as a function of PAI-1 residue number in (**b**). The per-residue average C_α_-RMSD values are indicated by the black line.

The irreversible transition of PAI-1 from active to latent form must be mechanistically preceded by reversible local fluctuations. We expect these fluctuations to be relatively infrequent, but of larger distance amplitudes. To search for such events we extracted the residue-specific C_α_-RMSD values from individual frames every 0.2 nanosecond of the four simulations on active PAI-1 and of the two simulations on latent PAI-1. These C_α_-RMSD values (one per frame) were then plotted as a histogram of C_α_-RMSD values grouped in bins of 0.1 Å (Fig. 5a). This representation clearly identifies residues with frequent low amplitude motions (*e.g*. S35) from residues where less frequent but larger amplitude motions occur (*e.g*. L69). To facilitate an evaluation of these traits on the global protein scale the histograms were depicted as a heat map along the amino acid sequence (Fig. 5b). A similar analysis was conducted on the simulation of latent PAI-1 and shown in Figure S7. Many regions, especially loops but also alpha-helical regions, show low frequency - large amplitude motions in the simulation of active PAI-1. The RCL as well as the sequence from residue 50 to 160 are some of the regions particularly influenced by potentially interesting large amplitude motions and we therefore investigated the simulation data from these regions in greater detail.

### Large collective RCL motions occur on the nanosecond timescale

In the first frame of the simulations on active PAI-1 the RCL is flipped towards the gate region (Figure S8a). As evaluated by the time-resolved distance between I342 in the RCL and K176 in the loop following β-strand 3A (Figure S8) the RCL appears to explore a range of conformations through large collective motions which appear rather stochastic. Insertion of the RCL into β-sheet A requires (at least) opening of the gap between β-strand 3A and 5A. We thus evaluated the distances between upper-most β3A and β5A hydrogen-bonded backbone nitrogen and carbonyl oxygen atoms as function of simulation time (Figure S8f and S8g). While the upper 2–3 hydrogen bonds appear to fluctuate between open and closed states the subsequent hydrogen bonds further down remain intact. Interestingly, application of 1×AMD boost does not seem to increase the propensity for gap formation. In none of the simulations is there any signs of preliminary insertion of RCL residues into β-sheet A, nor does β-strand 1 C detach from β-sheet C. Importantly, these simulations do not reproduce the extended gap between the entire length of β3A and β5A observed prior to RCL insertion in previous simulations of a forced transition to the latent form^18^.

### Dynamics in the W86-loop leads to progressive detachment of β2A from β3A

**Figure 6:**
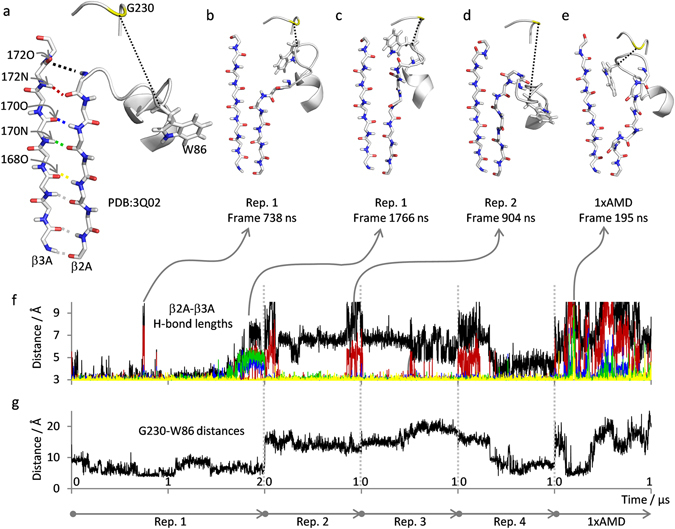
W86-loop dynamics leads to progressive detachment of β2A from β3A. Example structures of W86-loop-β2A-β3 A configurations are shown from (**a**) the PDB:3Q02 crystal used as basis for simulations and (**b**–**e**) from the indenticated frames in the simulations on active PAI-1. The W86 sidechain and β2A and β3 A backbones are shown as sticks and remaining residues as cartoon. Residue G230 is colored yellow and the distance between C_α_ atoms of G230 and W86 is indicated by the black broken line. Hydrogen-bonds between β2 A and β3 A are indicated by dashed colored lines as follows: 172 O:90 N (black), 172 N:90 O (red), 170 O:92 N (blue), 170 N:92 O (green) and 168 O:94 N (yellow). (**f**) β2A-β3A hydrogen-bond lengths, according to the color coding in (**a**), are plotted as a function of simulation time consecutively for all simulations on active PAI-1, as indicated. (**e**) the distances between C_α_ atoms of G230 and W86 are plotted as a function of simulation time consecutively for all simulations on active PAI-1, as indicated.

The W86-loop (connecting hD and β2A) is particularly influenced by large amplitude distance fluctuation in the simulation on active PAI-1 (Fig. 5b). To evaluate the temporal dynamics of the W86-loop we measured the distance between the C_α_-atoms of G230 and W86 as a function of simulation time (Fig. 6). Substantial changes in the W86-loop configuration, and in the orientation of the W86 sidechain, are observed. In the crystal structure of the W175F mutant of PAI-1^34^ the W86 sidechain is pointing out towards the solvent (Fig. 6a) whereas in the crystal structure of the 14-1B PAI-1 mutant it is pointing into the structure^35^. Interestingly, in the replicate 1 simulation of active PAI-1 (which is based on the crystal structure of W175F) the W86 sidechain starts pointing out, but flips into the structure after ~ 200 ns and stays pointing inwards for the remainder of that simulation (Fig. 6b,c and g). In replicate 2 and 3 the W86 sidechain is pointing outwards whereas in replicate 4 and the simulation with AMD boost it is fluctuating between inward and outward orientations (Fig. 6e,g). A number of substantially different conformations of the W86 loop were observed, as evidenced by the approx. 5–20 Å range of observed G230:W86 distances. Interestingly, detachment of β2A from β3A as a consequence of W86-loop dynamics was observed in some of the trajectories. We therefore monitored the distances between upper-most β3A and β2A hydrogen-bonded backbone nitrogen and carbonyl oxygen atoms as function of simulation time (Fig. 6f). These distances indicate that the upper hydrogen-bonds tethering β2A to β3A are progressively less stable than hydrogen-bonds further down. Indeed, the upper-most hydrogen-bond (172 O:90 N) is only formed in certain parts of certain simulations. Breakage of the upper two hydrogen bonds is observed in all replicate simulations of active PAI and in replicate 1 further hydrogen bonds are broken a couple of times during the simulation (Fig. 6b,c). Interestingly, application of the 1×AMD boost had a major impact on the stability of this region leading to numerous detachments of β2A from β3A (Fig. 6e,f). Taken together, these simulations indicate substantial dynamics of the W86-loop, with multiple different orientations of the W86 sidechain being sampled in solution, leading to occasional detachment of β2A from β3A.

### Helix F and helix D exhibits profound deformations during simulations

**Figure 7:**
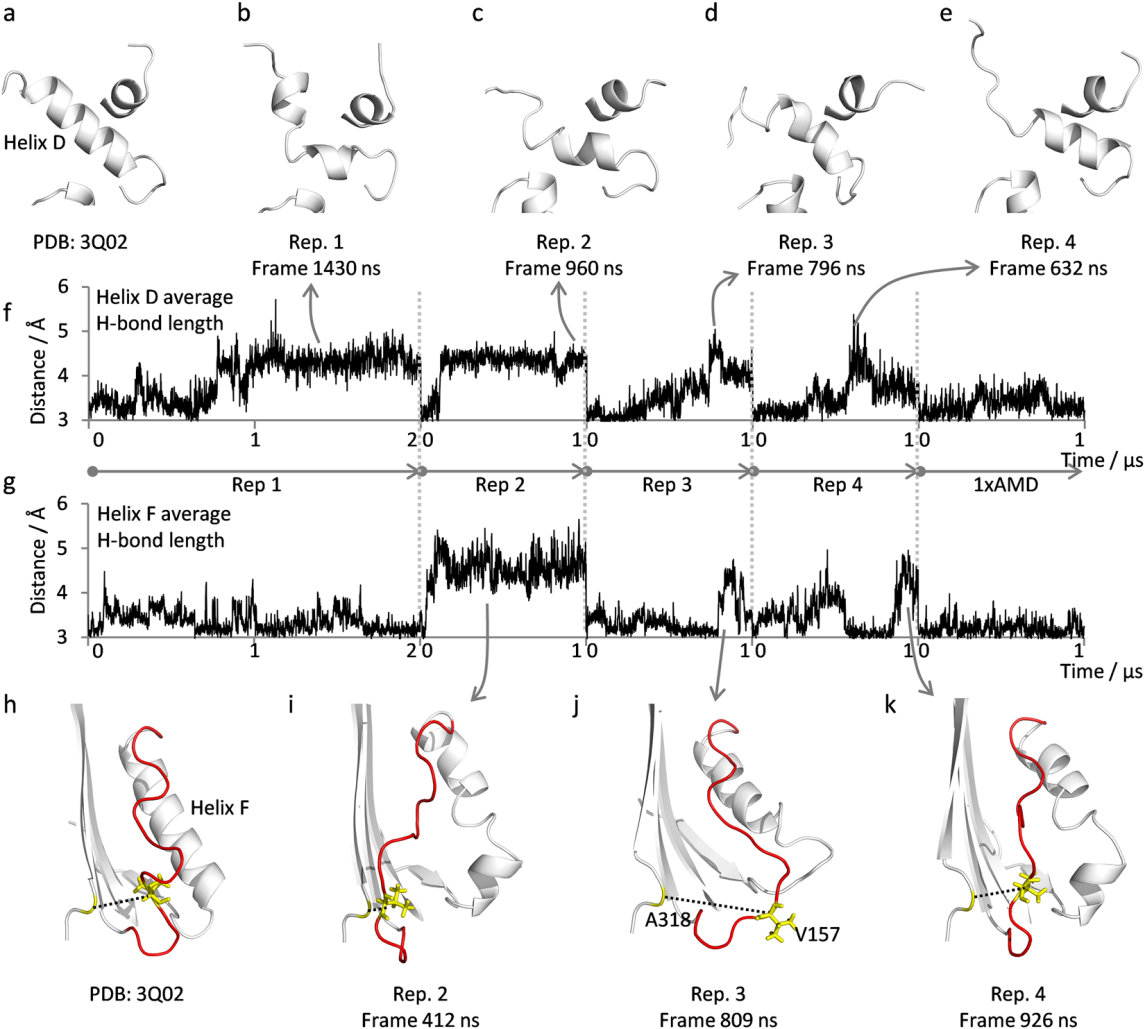
Helix D and F dynamics. Example structures of helix D and F configurations are shown in panels (**a–e** and **h–k**), respectively, from the indicated crystal structure and frames in the simulations on active PAI-1. Structures are generally shown as white cartoon representation with the exception of the loop between helix F and β-strand 3 A which is displayed in red. Residues V157 and A318 are colored yellow and the distance between the C_α_ atoms of these residues is illustrated by a black broken line and plotted as a function of simulation time in Figure S9. The average hydrogen bond lengths of helix D (all hydrogen bonds between 70 O:74 N and 79 O:83 N) and helix F (all hydrogen bonds between 128 O:131 N and 141 O:145 N) are plotted as a function of simulation time consecutively for all simulations on active PAI-1 in panel (**f** and **g**), respectively.

Both helix D and helix F are characterized by large amplitude fluctuations in the simulations on active PAI-1 (Fig. 5b). We have calculated the average hydrogen-bond length of all the hydrogen bonds defining these helices in the W175F PAI-1 crystal structure (PDB: 3Q02). As hydrogen bonds are generally shorter than 3.5 Å any average hydrogen bond length above this value implies that one or more of the bonds are broken. Significant increases in the average helix D hydrogen bond length are observed in all replicate simulations on active PAI-1 (Fig. 7a–f). The increased hydrogen bond lengths are caused by kinks, bends and helical unwinding at the termini as shown in Fig. 7a–e. Intriguingly, the average hydrogen bond length in the simulation with 1×AMD boost is not particularly increased in helix D (Fig. 7f), despite the dramatically increased C_α_-RMSF values (Figure S3c and S3e). Thus the entire helix D is moving relative to the average position but the helical integrity stays relatively unperturbed. A similar analysis of helix F also reveal increased average hydrogen bond lengths in simulations on active PAI-1 manifested as kinks and unwinding of helical turns, especially in the mid and lower part of helix F (Fig. 7g–k). Again, application of 1×AMD potential did not result in loss of helical integrity (Fig. 7g) despite significantly elevated C_α_-RMSF values (Figure S3c and S3e). The loop connecting the top of helix F with the bottom of β3 A (V157-loop) is also influenced by large amplitude distance fluctuation in the simulation on active wt PAI-1 (Fig. 5b) and generally assumes an array of different conformations in the simulations (Fig. 7h–k). To evaluate the time evolution of V157-loop dynamics we measured the distance between the C_α_-atoms of V157 and A318 as a function of simulation time (Figure S9). In the crystal structure of W175F PAI-1 the V157 sidechain is pointing into the structure and positioned in a hydrophobic pocket lined by F126, V129, L152, V164 and A318. In the simulation of active PAI-1 the V157 sidechain flips in and out of this pocket and these flips are associated with 5–7 Å changes in the distance between the C_α_-atoms of V157 and A318.

## Discussion

Conformational change underlines the function and dysfunction of serpins but the structural dynamics leading to conformational change in serpins is poorly understood. We recently discovered unanticipated native-state local unfolding in PAI-1^19^, a serpin renowned for its spontaneous conformational change to the inactive latent conformation. We now hypothesize that unfolding plays an important role in conformational change of serpins. To test this hypothesis, we will need to explore the extent, geometry and mechanism of unfolding and the role of unfolding for conformational change in serpins using advanced experimental and computational methods. While a number of serpins have previously been subjected to “free” molecular dynamics simulations in atomistic detail^26–31^, only simulations that artificially force the active to latent transition have to our knowledge previously been performed on PAI-1^18, 32^. We have conducted “free” molecular dynamics simulations on active and latent PAI-1 to unravel the inherent dynamics of this serpin at atomic resolution in the microsecond time scale.

Four replicate one-microsecond simulations were conducted on active PAI-1 and two replicate simulations on latent PAI-1. Evaluated on the basis of residue-specific C_α_-RMSF values (Figure S3) the reproducibility was generally very high among replicates, especially in the more stable regions of the molecule and thus in particular in the replicates of latent PAI-1. It is of course expected to observe greater variation among replicates in partially stable regions as the low frequency – large amplitude fluctuations are more likely to be undersampled. However, a general increase in the average C_α_-RMSD value with simulation time was observed in all replicate simulations on active PAI-1, indicating that one microsecond is not enough to completely equilibrate active PAI-1 to the simulation conditions. This is expected as one microsecond is still a very short period of time, but also considering the fact that active PAI-1 is a metastable conformation at the simulated conditions and eventually should convert to the latent form, if the simulations are realistic. A free simulation of PAI-1 in the active conformation is therefore expected not to reach equilibrium. However, as is evident from the presented data and as we discuss further below, there are some well-defined dynamic hot spots in the active PAI-1 structure that we have identified with our simulations.

Evaluation of residue-specific C_α_-RMSF values revealed high structural flexibility in the RCL and in alpha-helices and connecting loops in most of the lower half of the serpin whereas especially the central parts of β-sheet A and β-sheets B and C were much more rigid (Fig. 2b). Interestingly, the pattern of flexible and rigid regions obtained through these simulations correlate well with the pattern previously obtained through hydrogen/deuterium-exchange experiments at 25 °C or 37 °C^19, 21, 22^. This is not surprising, as structural flexibility is often associated with solvent exposure and transient breaking of backbone hydrogen bonds, - the two prerequisites for hydrogen/deuterium-exchange on backbone amides. Nevertheless, many of the most flexible regions of PAI-1 acquire full deuteration prior to the first labeling time-point at 25 °C or 37 °C, which renders HDXMS at these temperatures unable to resolve the kinetics of the fastest dynamics. To explore the scope of our simulations further, we therefore conducted an HDXMS experiment at 5 °C that would slow down and resolve fast dynamics better than our previous experiments. Interestingly, the exchange data acquired at 5 °C correlate very well with our simulation data and thus supports the validity of the trends observed in the simulations. Although a correlation between HDXMS data and C_α_-RMSF values obtained from molecular dynamics simulation is expected there are important differences to keep in mind and also reasons why a perfect correlation should not be expected. HDXMS and simulation data are acquired at widely diverse timescales (10^7^–10^9^ fold difference) and there is an extreme difference in the number of analyzed molecules (1 vs. 2.4 × 10^13^). The structural flexibility facilitating hydrogen deuterium exchange may thus be grossly undersampled in the simulations and therefore not reflected in C_α_-RMSF values. Only a subset of the local structural fluctuations monitored by simulation C_α_-RMSF values can lead to backbone NH exchange. First, the protecting hydrogen bond must break and the H-bond donor or acceptor atom must be displaced by at least 3 Å to allow access to the catalyst ions (*i.e*.hydroxide or deuteroxide anion)^36, 37^. Furthermore, exchange kinetics depends strongly on the life time of the unprotected state.Allister and Konermann recently explored the correlation between experimental backbone NH exchange and the predicted exchange behavior based on MD simulations of ubiquitin. Although the exchange behavior of the majority of NHs was in excellent agreement with the MD simulation results, a minority of NHs exhibited an exchange behavior that did not seem to correlate with predicted exchange based on MD simulations^38^. Nevertheless, it is expected that regions characterized by large C_α_-RMSF values at very short timescales would also be prone to breaking of backbone hydrogen bonds and solvent accessibility at longer timescales. An exception to such a correlation would of course be rigid domain motions, where a larger group of hydrogen bonded residues move together relative to other domains of the protein. In this scenario large C_α_-RMSF values may be high while NH exchange rates remain low. However, PAI-1 is a globular protein without sub-domains and therefore not likely to exhibit such a behavior.

Considerable interest has previously been devoted to the integrity of β-sheet A and especially the gap between the apex of β-strand 3A and 5A during molecular dynamics simulations. The prevailing model for insertion of the RCL into β-sheet A is that it occurs in a zipper-like fashion where β-strand 3A and 5A separates from the top down in-sync with the insertion of more and more RCL residues. In support of this model, it has been noted that serpin mutants especially prone to polymerization through β-sheet A expansion exhibits a larger gap between these β-strands than their wild-type counterparts in nanosecond molecular dynamics simulations^26, 28^. Although the formation of a small gap between β-strand 3A and 5A was observed in our simulations (Figure S8g) we did not see any sign of insertion of RCL residues into the gap. Complete separation of β-strand 3A and 5A followed by insertion of the RCL, without significant unfolding of any other secondary structure, was previously observed in a targeted simulation on PAI-1 where latency transition is forced^18^. The small gap observed in the apex of β-sheet A did not propagate further down in our simulations, which is somewhat expected as surrounding structures were still completely intact. Clearly, the “free” simulations presented here are much too short to realistically probe the dynamics leading all the way to the latent state. However, our simulations do not provide new support for the idea that opening of a small gap in the apex of β-sheet A is sufficient for RCL insertion.

Of particular relevance for the proposed hypothesis of a role of unfolding in PAI-1 latency transition, we observed substantial dynamics in helix C, the entire flexible joints region, helix F and the following loop (residues 50–160). A correlation between instability in helix B and C and PAI-1 latency transition was previously observed^21, 22^ and unfolding was detected on the seconds time-scale using hydrogen/deuterium-exchange^19^. Although helix C generally remained intact in simulations, relatively high C_α_-RMSF values were observed in this region in some simulations. Interestingly, fluctuations in the W86-loop lead to progressive detachment of β-strand 2A from β-sheet A in certain instances (Fig. 6). Instability in the “flexible joints” region has been suggested to play an important role in the conformational change of PAI-1 based on the observation that many ligands which moderate the rate of latency transition, and/or induce substrate behavior or polymerization in PAI-1, actually bind in this region^21–25^. The simulations conducted here provide new detailed insights to the dynamics of the W86-loop and suggests that this loop and the following β-strand 2A may act as a site of “first breach” in the PAI-1 structure. Major fluctuations were also observed in the V157-loop in our study. A remarkably slow rate of latency transition is observed in the 14-1B mutant of PAI-1 (~145 fold slower than wild-type)^39^. This mutant exhibits a mutationally-induced 3_10_ helix in the V157-loop^35^, which likely stabilizes this region. Taken together, these observations argue for a role of V157-loop plasticity in the mechanism of latency transition.

As helix F and the V157-loop obstruct RCL insertion into β-sheet A, these structures somehow need to move away or unfold for the RCL to insert. Following this line of argument it is interesting that regions on either side of helix F (the V157-loop and the W86-loop) are extremely dynamic in the active form of PAI-1. Occasionally, these instabilities may spread to surrounding structures in the flexible joints region such as helix F and helix D. A progressively destabilized and perhaps ultimately unfolded flexible joint region on the micro to millisecond timescale may indeed facilitate the previously measured unfolding in helix B and C at the second timescale. Unfolding of lower helix B and C may in-turn lead to progressive unfolding of the hydrophobic core and β-sheet A on the minute time-scale. The observed detachment of β-strand 2A in these simulations provide new support for the idea that β-sheet A may substantially unfold prior to latency transition. In conclusion, the simulations conducted here therefore provide new support for the conjecture that latency transition may occur as the result of alternative refolding from an unfolded state, rather than as a zipper-like insertion of the RCL into a minimally expanded β-sheet A. Furthermore, our simulations increase the understanding of the structural dynamics in serpins at the shortest timescales and at atomic level and show an interesting correlation with HDX data. We thus believe that further simulations, including steered or biased dynamics, will prove valuable in elucidating the mechanism of conformational change in serpins in the future.

## Methods

### Molecular dynamics simulation of active and latent PAI-1

“Free” atomistic molecular dynamics simulations of active and latent PAI-1 were conducted using the AMBER 14 software suite^40^. As no crystal structure of human wild-type PAI-1 in the active conformation is available, we have used the crystal structure of the W175F mutant as a basis (PDB: 3Q02)^34^. Missing RCL residues as well as the F175W amino acid substitution back to wild-type were modelled-in using Modloop^41, 42^. The crystal structure PDB: 1DVN^35^ was used for simulations on PAI-1 in the latent conformation. Input parameters and topology files for MD simulations were created using xLeap and the ff99SB force field. The proteins were solvated in a truncated octahedron box of TIP3P water with a minimum distance of 10 Å between all protein atoms and the edge of the water box and the system was neutralized by addition of Na^+^ ions. Periodic boundary conditions were used. The protein systems were subjected to a five-step minimization and equilibration regime prior to production MD simulations as follows: ***1)*** Minimization of solvent and ions (protein fixed) using 500 steps of steepest decent followed by 500 steps of conjugate gradient. ***2)*** Minimization of the whole system using 1000 steps of steepest decent and 1500 steps of conjugate gradient. ***3)*** 20 ps of MD simulation with heating from 10 K to 310 K, constant volume and weak temperature coupling^43^ on the whole system and weak positional restraints on the protein using 1 fs time steps. SHAKE^44^ was used to constrain bonds involving hydrogen and the Particle Mesh Ewald summation method^45^ was used for calculation of electrostatic energies with a 10 Å cutoff of non-bonded interactions. ***4)*** 10 ps of MD simulation with the same settings as in step ***3)*** but at a constant temperature of 310 K (37 °C). ***5)*** 50 ps of MD simulation as in step ***4)***, but with 2 fs time steps, no restrains on the protein and with constant pressure at 1 bar maintained with the Berendsen barostat^43^. Production simulations were conducted for 1 µs (or 2 µs) with the same parameters as for step ***5)*** of the equilibration, apart from a non-bonded cut-off of 9 Å. Energies and coordinates were written every 1000 steps (2 ps) for subsequent analysis of the simulation trajectories. Four replicate 1 µs simulations were conducted for PAI-1 in the active conformation and two replicate 1 µs simulations were conducted on PAI-1 in the latent conformation. Non-identical initial atom velocities in replicate experiments was secured by heating from 10 K rather than 0 K. Additionally, one of the 1 µs simulations of active PAI-1 was extended to 2 µs and two additional 1 µs simulation were conducted with either 1× or 2× AMD boost to the potential energy function. Boost values were determined based on the average total potential energy (−142296 kcal/mol) and average dihedral potential energy (4105 kcal/mol) obtained from a short conventional MD simulation, as well as the total number of atoms in the system (48090) and the number of residues in the protein (379) as described in^33^. Boost parameters were set as follows: 1×AMD (iamd = 3, EthreshD = 5431.5, alphaD = 265.3, EthreshP = −134602, alphaP = 7694.4), 2×AMD (iamd = 3, EthreshD = 6758, alphaD = 265.3, EthreshP = −126907, alphaP = 7694.4). All calculations were conducted using the GPU-enabled version of PMEMD^46, 47^ on a custom-build AMBER-certified workstation equipped with four GeForce GTX 980 GPU’s (EXXACT Corporation). Trajectories were analyzed with cpptraj^48^ and visualized with VMD. Renditions of simulated structures were made with The PyMOL Molecular Graphics System, Version 1.5 (Schrödinger, LLC.)

### Chemicals and Materials

D_2_O (99.9 atom % D) was obtained from Cambridge Isotope. Deuterated guanidine HCl (98 atom % D) was from Sigma-Aldrich. Pepsin immobilized on agarose beads was from Pierce. Recombinant human active and latent PAI-1 was produced in *E. coli*.

### Hydrogen/deuterium-exchange experiments

40 pmol (0.2 µM) active or latent PAI-1 was allowed to undergo isotopic exchange in 99% deuterated buffer containing 10 mM phosphate, 137 mM NaCl, pD 7.4 at 5 °C for 1, 5 or 20 minutes. Triplicate measurements of these time points were made. Active PAI-1 was additionally subjected to 0.5, 2, 15, 30, 40 and 80 minute incubation time points. The exchange reactions were quenched by addition of formic acid to a final concentration of 0.6% and snap-frozen in liquid nitrogen. Fully deuterated PAI-1 control was prepared by incubation of 40 pmol active PAI-1 in 7.2 M deuterated guanidine HCl for 5 min at room temperature followed by 9.5-fold dilution in D_2_O with 1% formic acid. Online proteolysis and LC-MS analysis of both undeuterated and deuterated PAI-1 samples was done using a cooled nanoACQUITY UPLC system with HDX technology coupled to a Synapt G2 mass spectrometer (Waters). The samples were injected into a loop wherefrom they were flushed through a 2 × 5 mm column packed with immobilized pepsin (Pierce) and desalted on an ACQUITY UPLC BEH C18 VanGuard Pre-column, (130 Å, 1.7 µm, 2.1 × 5 mm, Waters) using a solution of 0.23% formic acid at 500 µL/min for 1.5 minutes. The peptides were eluted off the precolumn and separated on a ACQUITY UPLC BEH C18 (300 Å, 1.7 µm, 1 × 50 mm, Waters) analytical column using a gradient of solvent A (0.23% formic acid) and solvent B (acetonitrile + 0.23% formic acid) from 15–30% B in 4 minutes and from 30–90% in 0.5 minutes at a flow of 50 µL/min. The mass spectrometer was operated with a capillary voltage of 3.00 kV, cone voltage of 20 V, extraction cone voltage of 3.6 V, ion source block temperature at 100 °C with a desolvation gas flow of 1000 L/h at 250 °C. Peptic peptides from non-deuterated active PAI-1 were identified using data-independent MS/MS acquisition (Waters MS^E^) as well as data-dependent MS/MS acquisition of the 3 most intense peptide signals in each MS spectrum.

### Hydrogen/deuterium-exchange data analysis

Raw mass spectral data of undeuterated samples were processed using PLGS 2.5 (Waters) and searched against a custom made database including the PAI-1 sequence for identification of peptic peptides using PLGS 2.5 (Waters) or MASCOT (Matrix Science). A total of 103 peptides were identified, covering 99% of the PAI-1 sequence. Extraction of MS spectra of relevant peptides and calculation of peptide average masses was done using DynamX (Waters). Calculation and plotting of deuterium contents, as well as preparation of relative deuterium heat maps, were done in Excel (Microsoft). Identification of overlapping peptide sequences allowed the calculation of deuterium uptake on the overlapped residues by subtraction of deuterium content and referred to as “sub-peptides”. This method requires the back-exchange level of the two peptides used to calculate the deuterium content of sub-peptides to be comparable^49^. The average deviation in back-exchange between the peptide pairs used to produce the sub-peptides is 2.7%-points and the maximum was 8.2%-points. We find this error to be negligible and without implications for the interpretation of the data. A subset of 38 peptides and sub-peptides, which gave the optimal coverage of the full PAI-1 sequence, were utilized in the preparation of a relative (to the full deuteration level) deuteration heat map (Fig. 3). Crystal structure images and models were prepared using The PyMOL Molecular Graphics System, Version 1.5 (Schrödinger, LLC.).

### Data Availability

The datasets generated during and/or analysed during the current study are available from the corresponding author on reasonable request.

## Electronic supplementary material

Supplementary Information
