# Supplementary material for: Conformational preludes to the latency transition in PAI-1 as determined by atomistic computer simulations and hydrogen/deuterium-exchange mass spectrometry
Source: Sci Rep. 2017 Jul 26;7:6636. doi: 10.1038/s41598-017-06290-0 (PMC5529462; doi:10.1038/s41598-017-06290-0)
Supplement: Supplementary file 1 — Supplementary Information [file 41598_2017_6290_MOESM1_ESM.pdf]

Supplementary Information

for

Conformational preludes to the latency transition in PAI-1 as determined by atomistic computer simulations and hydrogen/deuterium-exchange mass spectrometry

by

Michael Petersen[1], Jeppe B Madsen[2], Thomas JD Jørgensen[2] and Morten B Trelle[2]*

[1] Department of Physics, Chemistry and Pharmacy, University of Southern Denmark, Campusvej 55, 5230, Odense M, Denmark
[2] Department of Biochemistry and Molecular Biology, University of Southern Denmark, Campusvej 55, 5230, Odense M, Denmark

*Corresponding Author
Morten Beck Trelle (morten.beck.trelle@rsyd.dk)
Affiliation as of January 1. 2017: Svendborg Hospital, OUH, Baagøes Alle 15, 5700, Svendborg, Denmark

Contents of this Supplementary Information:

Page 2: Notes to Supplementary Figures
Page 3: Figure S1: Structural stability of PAI-1 during molecular dynamics simulations.
Page 4: Figure S2: Structural stability of PAI-1 during molecular dynamics simulations.
Page 5: Figure S3: Localized microsecond dynamics from PAI-1 molecular dynamics simulations.
Page 6-9: Figure S4: Hydrogen/deuterium-exchange data of active and latent PAI-1.
Page 10: Figure S5: Hydrogen/deuterium-exchange data of latent PAI-1.
Page 11: Figure S6: Comparison of simulation C_α-RMSF values with hydrogen/deuterium-exchange data.
Page 12: Figure S7: Low frequency – large amplitude motions in latent PAI-1.
Page 13: Figure S8: RCL and β5A and β3A dynamics in active PAI-1.
Page 14: Figure S9: V157-loop dynamics in active PAI-1.

Notes to supplementary Figures:

Supporting results : To evaluate the overall stability of the simulations we computed C_α-RMSD values relative to the first frame of the simulation every 0.2 nanosecond and averaged them over all residues or over all residues except the RCL and residues 50-160 ***(Figure S1)***. The C_α-RMSD value averaged over all residues and all frames of the simulation is 3.20 ± 0.52 Å for the active PAI-1 simulations and 2.03 ± 0.06 Å for the latent simulations ***(Figure S1j)***. This difference suggests substantial contribution of RCL dynamics to the high average C_α-RMSD value of active PAI-1 due to the surface exposed loop conformation ***(Figure 1a)***. Exclusion of RCL residues 229-249 reduced this average C_α-RMSD value of active PAI-1 to 2.17 ± 0.23 Å and further exclusion of residues 50-160, encompassing helix C and D, the flexible joints region, helix F and the following loop, reduced the average C_α-RMSD further to 1.61 ± 0.14 Å ***(Figure S1j)***. The regions of instability contributing to the higher average C_α-RMSD in active PAI-1 compared to latent is thus primarily the RCL. Exclusion of residues 50-160 reduced the C_α-RMSD value further in active PAI-1, but not in latent PAI-1, indicating that other parts of latent PAI-1 is contributing high C_α-RMSD values. An increase in the C_α-RMSD value of active PAI-1 is observed as a function of simulation time ***(Figure S1a and S1b)***, indicating a rather slow and incomplete equilibration to the simulation conditions, whereas only a very modest increase is observed for latent PAI-1 ***(Figure S1d and S1e)***. While the simulation of active PAI-1 with 1x AMD boost displayed a slightly increased but stable C_α-RMSD value in the last 500 ns of the simulation, the simulation with 2xAMD boost was clearly unstable and therefore not analyzed further ***(Figure S1g, S1h and S1i)***. Extension of the simulation time to 2 microseconds for replicate 1 did not indicate a continued increase in the average C_α-RMSD, suggesting that a local energy minimum is reached in this simulation ***(Figure S1g, S1h and S1i)***. The average structures from each replicate simulation of active PAI-1 align nicely in the majority of the active PAI-1 structure ***(Figure S2b)***, although a few select regions showed considerable variation. This included the RCL, helix D, the loop to β-strand 2A, helix E, I and F and the following loop region ***(Figure S2b-2e)***. We also computed the combined average structure of the replicate simulations and compared it to the crystal structure used as a basis for the simulation. Shifts of helix C and E as well as rotations of helix D and F relative to the crystal structure are clearly observed ***(Figure S2g-2i)***. These data suggest that the areas around helix I, C, D, the flexible joint region and helix F are quite flexible and that the structures populated in solution are perturbed relative to the crystal structure.

Figure S1

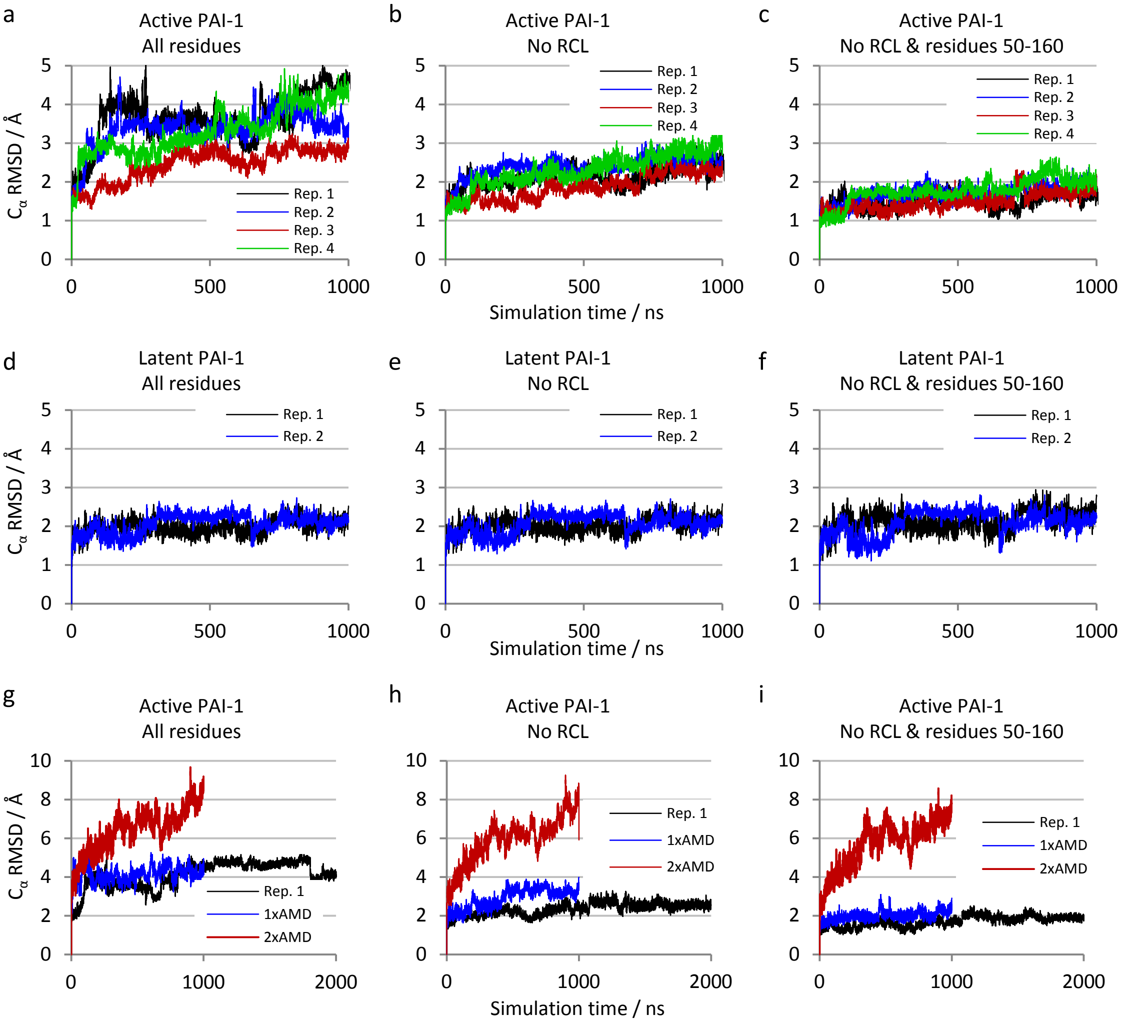

j

0-1000 ns simulations	Average C_α-RMSD ± SD (Å) All residues	All residues (- RCL)	All residues (- RCL and 50-160)
Active PAI-1 (all frames)	3.20 ± 0.52 (n=4)	2.17 ± 0.23 (n=4)	1.62 ± 0.14 (n=4)
Active PAI-1 (last 100 ns)	3.75 ± 0.80 (n=4)	2.53 ± 0.25 (n=4)	1.84 ± 0.17 (n=4)
Latent PAI-1 (all frames)	2.03 ± 0.06 (n=2)	2.05 ± 0.03 (n=2)	2.11 ± 0.03 (n=2)
Latent PAI-1 (last 100 ns)	2.12 ± 0.01 (n=2)	2.13 ± 0.06 (n=2)	2.23 ± 0.12 (n=2)
1xAMD active PAI-1 (all frames)	4.12 (n=1)	2.89 (n=1)	1.99 (n=1)
1xAMD active PAI-1 (last 100 ns)	4.13 (n=1)	3.23 (n=1)	2.27 (n=1)
2xAMD active PAI-1 (all frames)		5.91 (n=1)	
2xAMD active PAI-1 (last 100 ns)		7.71 (n=1)	
**0-2000 ns simulation**			
Active PAI-1 (all frames)	4.11 (n=1)	2.34 (n=1)	1.71 (n=1)
Active PAI-1 (last 100 ns)	4.12 (n=1)	2.54 (n=1)	1.91 (n=1)

Figure S1: Structural stability of PAI-1 during molecular dynamics simulations. The average RMSD over all C_α atoms relative to the first frame of the simulations were calculated once every 200 ps and plotted as a function of simulation time for ***a-c)*** the four replicate simulations of active PAI-1, ***d-f)*** the two replicate simulations of latent PAI-1 and ***g-i)*** the two-microsecond simulation of active PAI-1 (rep. 1) as well as the simulations on active PAI-1 with 1x and 2x AMD boost to the potential energy function. In ***a)***, ***d)*** and ***g)*** the C_α-RMSD values are averaged over all residues in PAI-1. In ***b)***, ***e)*** and ***h)*** the C_α-RMSD values are averaged over all residues in PAI-1, except RCL residues 229-249. In ***c)***, ***f)*** and ***i)*** the C_α-RMSD values are averaged over all residues in PAI-1, except RCL residues 229-249 and residues 50-160 (covering helix C, D, β-strand 2A, helix E, F and the following loop). ***j)*** Tabulated average C_α-RMSD values over all frames or over the last 100 ns of the simulation. The values are calculated as averages over replicates ± the standard deviation (where n > 1).

Figure S2

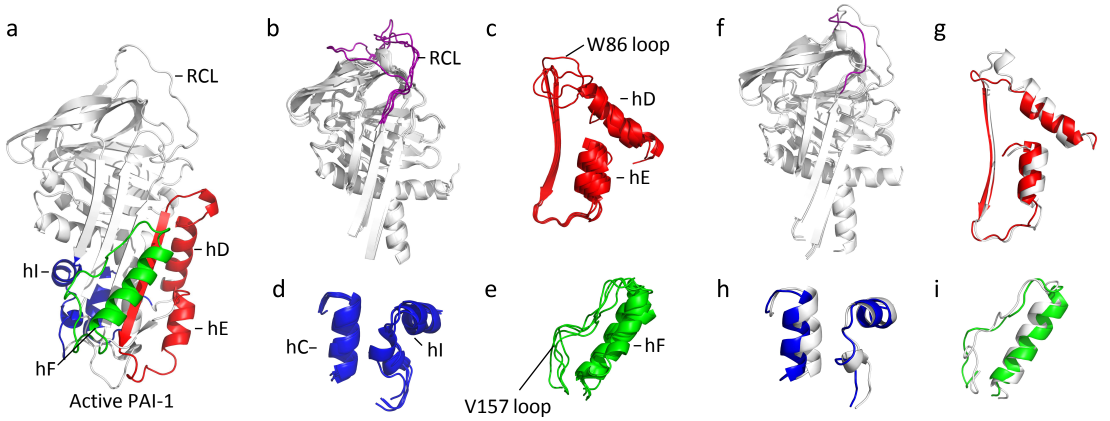

Figure S2: Structural stability of PAI-1 during molecular dynamics simulations. ***a)*** crystal structure of active PAI-1 (PDB: 3Q02) with missing RCL residues and the F175W mutation back to wild-type modelled-in using Modloop [38, 39]. Structural regions shown in subsequent panels are color coded for reference. Average structures from each replicate one-microsecond simulation on active PAI-1 are aligned and structral regions of this alignment is shown in panels ***b-e)*** to emphasize the areas where variation is most pronounced. The RCL is shown in purple in ***b)***. The average of the replicate average structures was aligned with the parent crystal structure in panels ***f-i***). Both the simulation average structure and the crystal structure are colored white in ***f)***, apart from the RCL which is colored purple in the simulation average structure. In ***g-i)*** the crystal structre is colored white and the simulation average structure are colored red, blue and green, respectively.

Figure S3

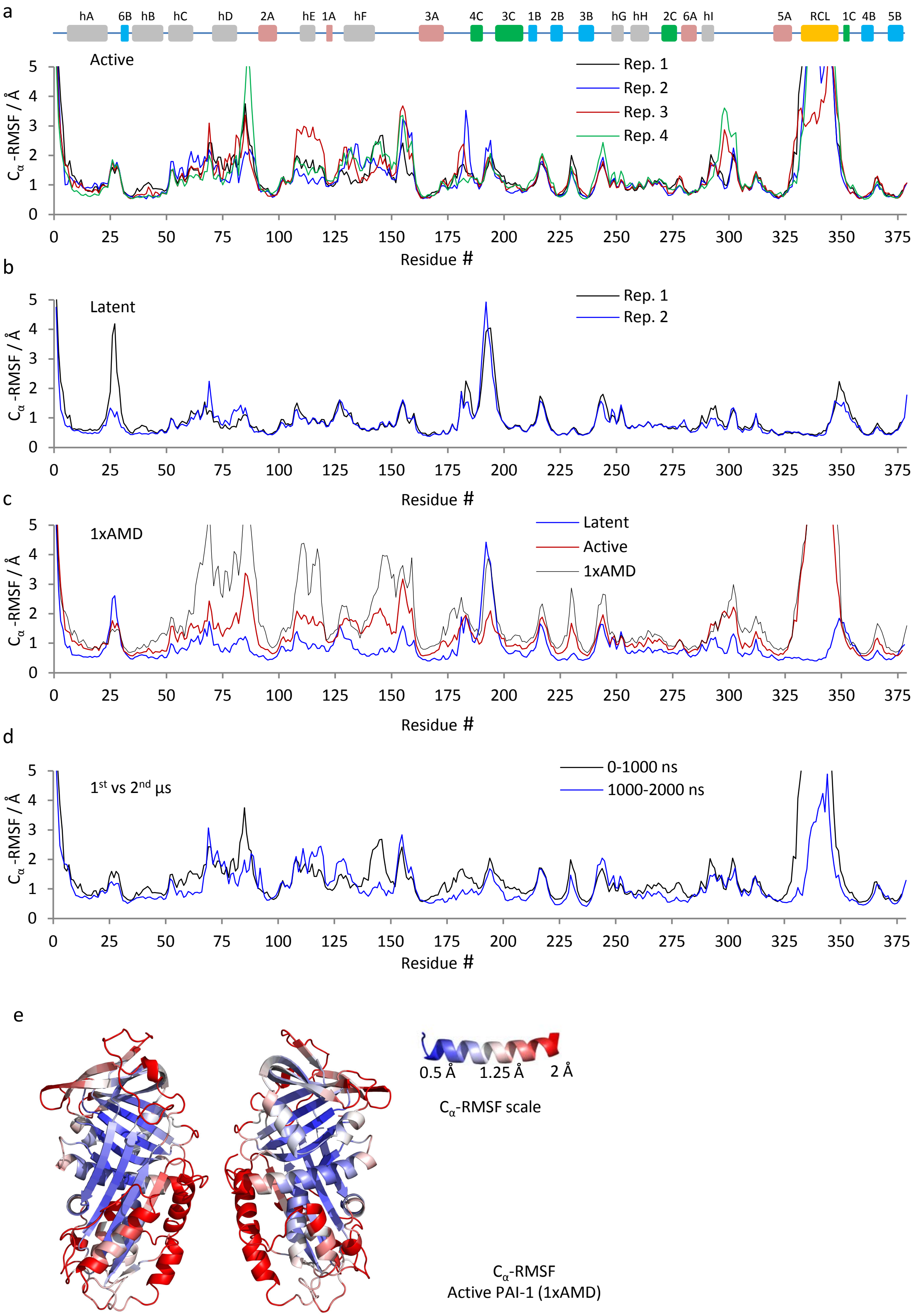

Figure S3: Localized microsecond dynamics from PAI-1 molecular dynamics simulations. The C_α-RMSF values from each of the four replicate one-microsecond simulations on ***a)*** active PAI-1 and the two one-microsecond simulations on ***b)*** latent PAI-1 are shown. Secondary structural elements are depicted above the plot for reference. The RCL is colored yellow, α-helices are colored grey, β-strands from β-sheet A, B and C are colored red, blue and green, respectively. ***c)*** The C_α-RMSF values from the simulation of PAI-1 with 1xAMD boost added to the potential energy function is shown together with average C_α-RMSF values of the replicates for active and latent PAI-1. ***d)*** C_α-RMSF values from 0 to 1000 ns and from 1000-2000 ns of rep. 1 of the active PAI-1 simulation. ***e)*** The average C_α-RMSF values from the simulation on active PAI-1 with 1xAMD boost to the potential energy function is indicated on the structure of active PAI-1 according to the indicated color scale.

Figure S4

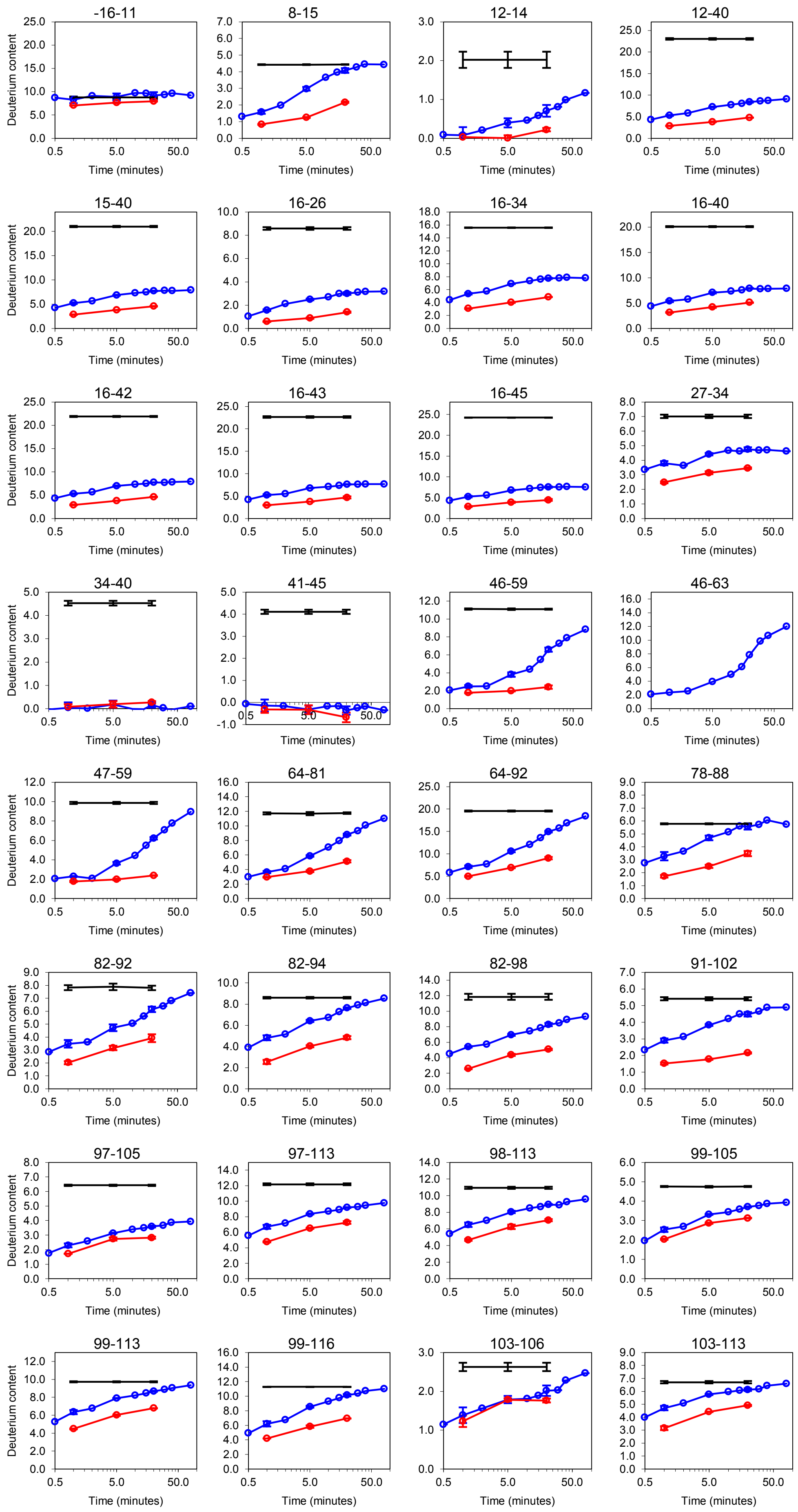

-16-11
8-15
12-14
12-40
15-40
16-26
16-34
16-40
16-42
16-43
16-45
27-34
34-40
41-45
46-59
46-63
47-59
64-81
64-92
78-88
82-92
82-94
82-98
91-102
97-105
97-113
98-113
99-105
99-113
99-116
103-106
103-113
Deuterium content
Time (minutes)

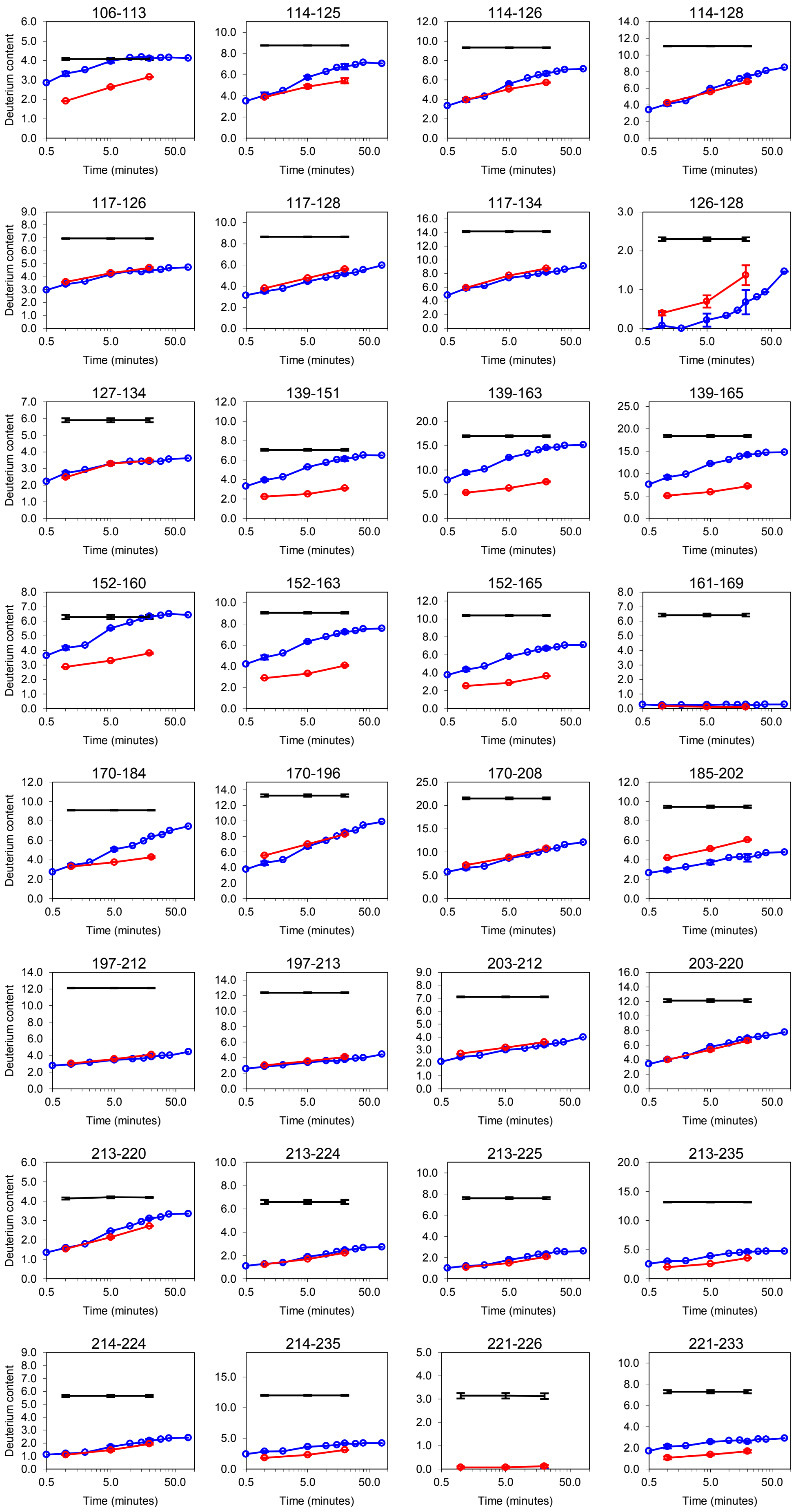
106-113
114-125
114-126
114-128
117-126
117-128
117-134
126-128
127-134
139-151
139-163
139-165
152-160
152-163
152-165
161-169
170-184
170-196
170-208
185-202
197-212
197-213
203-212
203-220
213-220
213-224
213-225
213-235
214-224
214-235
221-226
221-233
Deuterium content
Time (minutes)

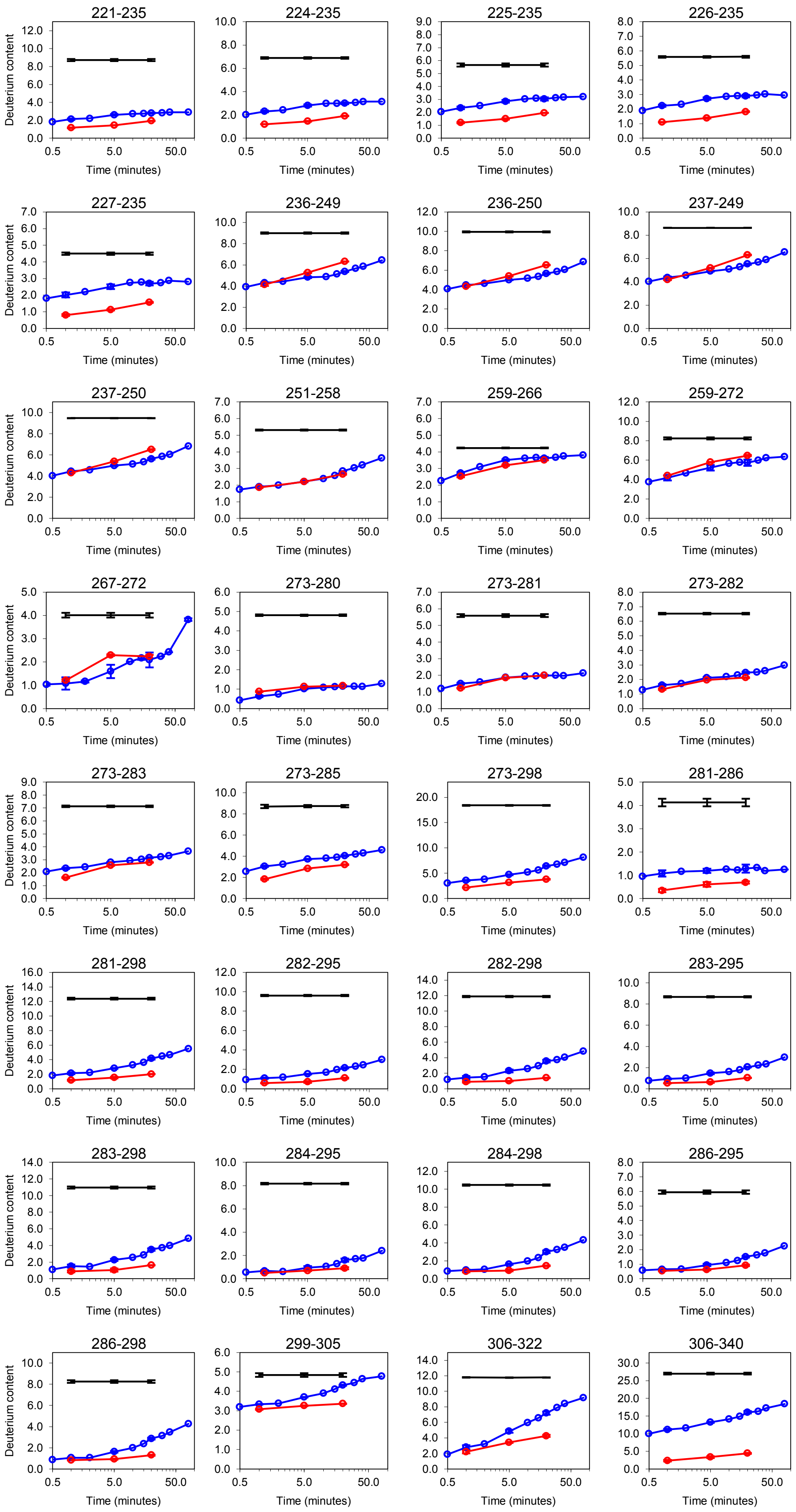
221-235
224-235
225-235
226-235
227-235
236-249
236-250
237-249
237-250
251-258
259-266
259-272
267-272
273-280
273-281
273-282
273-283
273-285
273-298
281-286
281-298
282-295
282-298
283-295
283-298
284-295
284-298
286-295
286-298
299-305
306-322
306-340
Deuterium content
Time (minutes)

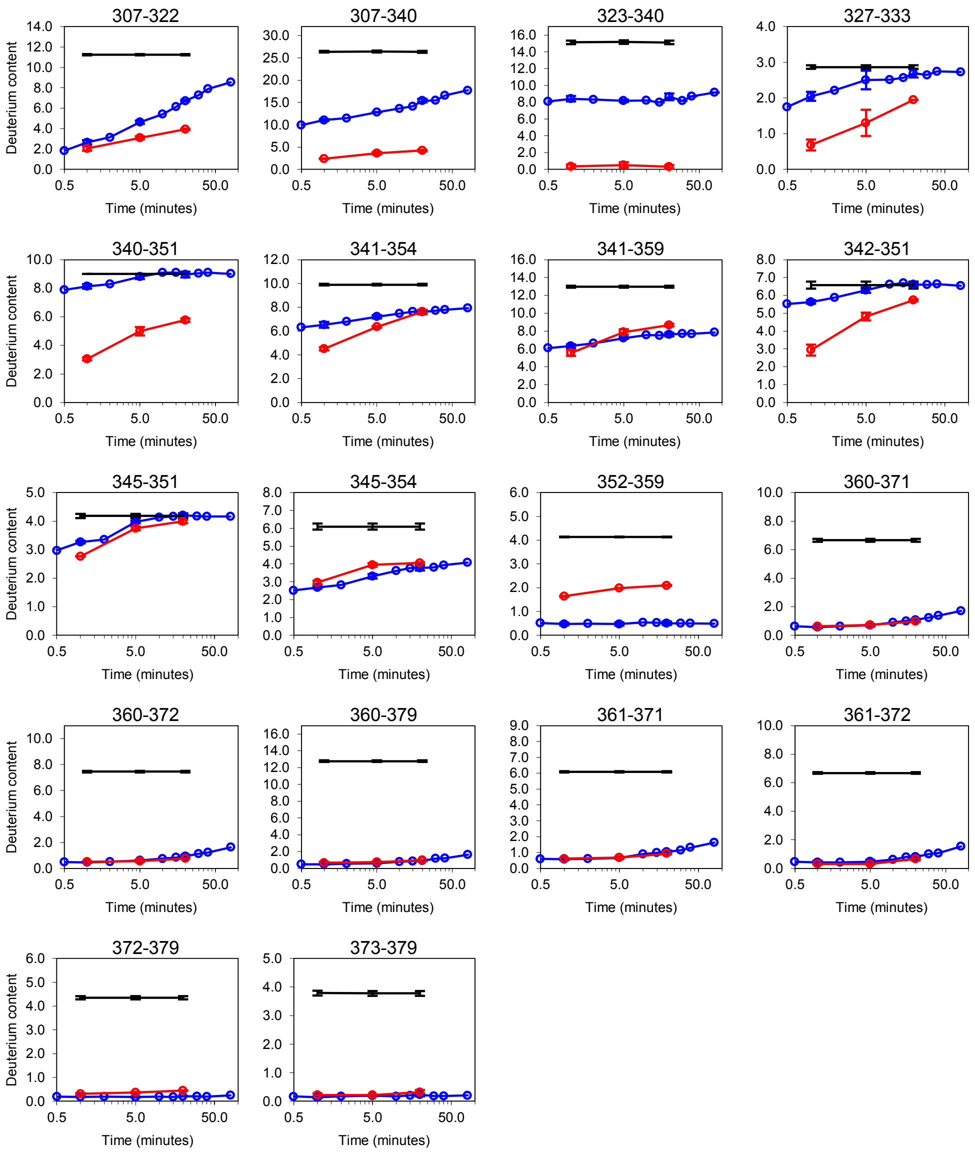

Figure S4. Hydrogen/deuterium exchange data of active and latent PAI-1. Deuterium uptake plots of the indicated peptides. Active and latent PAI-1 are indicated by the blue and red line, respectively, and the full deuteration level by the black line. Error bars represent the standard deviation from triplicate measurements.

Figure S5

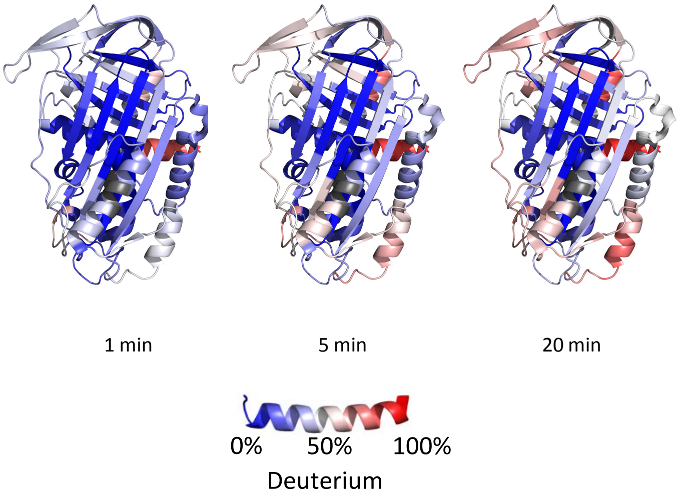

Figure S5: Hydrogen/deuterium-exchange data of latent PAI-1. The heat map of relative deuterium content from ***Figure 3a*** is mapped on the structure of latent PAI-1.

Figure S6

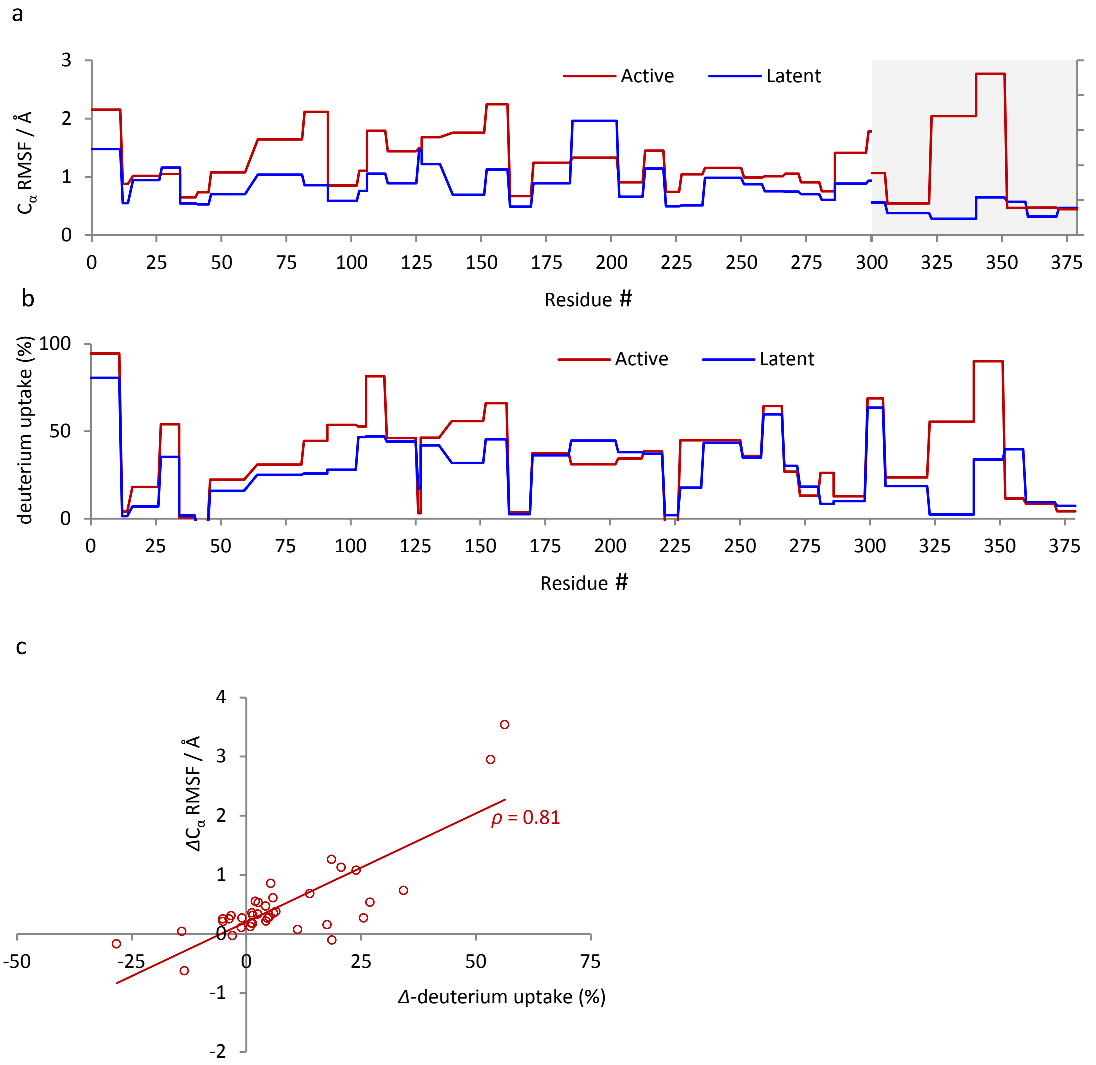

Figure S6: Comparison of simulation C_α-RMSF values with hydrogen/deuterium-exchange data. ***a)*** Amino acid specific C_α-RMSF values ***(from Figure 2a)*** averaged for the residues in each of the peptides analyzed in the HDX experiment is plotted for active and latent PAI-1**.** Note the change of scale for residues 300-378 (grey area). ***b)*** Relative deuterium uptake after 1 minute exposure to D_2O of active and latent PAI-1. The correlation between C_α-RMSF values and relative deuterium uptake for active (red) and latent (blue) peptides is investigated by plotting the two data sets on each axis and shown in ***Figure 4*** of the main article. ***c)*** Differences between C_α-RMSF values for active and latent PAI-1 were plotted against differences between relative deuterium uptake for active and latent PAI-1. The trendline and Pearson correlation coefficient ρ is shown.

Figure S7

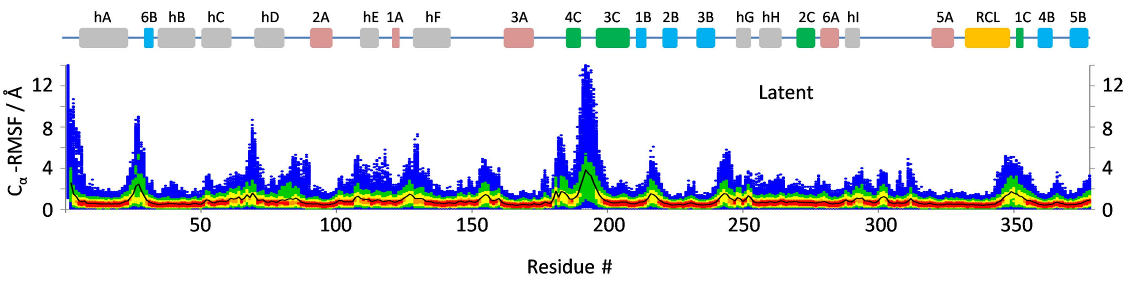

Figure S7: Low frequency – large amplitude motions in latent PAI-1. The per-residue, per-frame C_{α}-RMSD values relative to the average structure was calculated once every 200 ps for the two simulations on latent PAI-1 (total of 10000 frames) and plotted as in ***Figure 5b***.

Figure S8

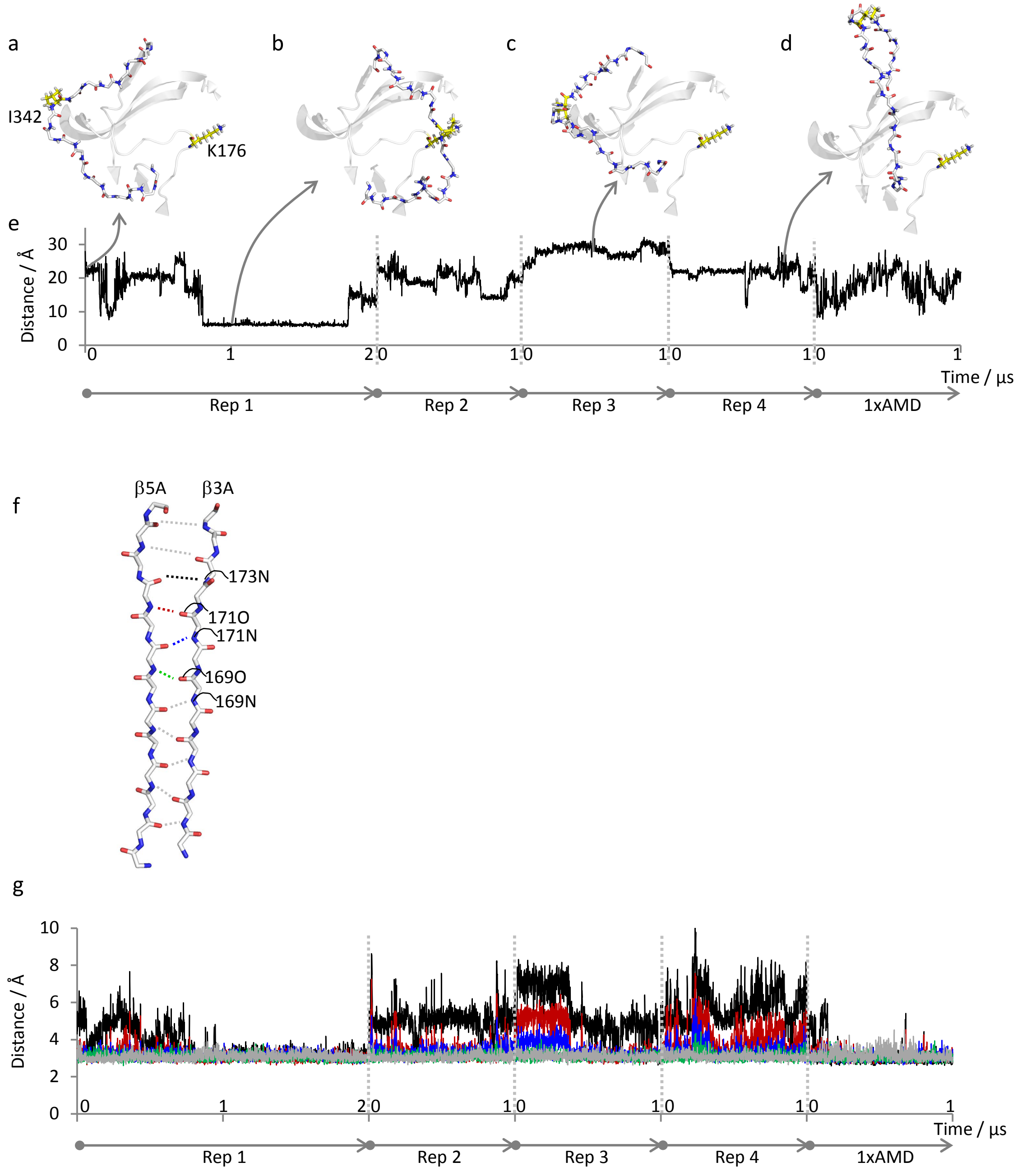

Figure S8: RCL and β5A and β3A dynamics in active PAI-1. Snap-shot structures of RCL configurations in the simulations of active PAI-1 from ***a)*** the first frame of Rep 1, ***b)*** the 1 µs frame of Rep 1, ***c)*** the 0.5 µs frame of Rep 3 and ***d)*** the 748 ns frame of Rep 4. The backbone of RCL residues (aa 330-350) are shown as sticks and parts of the underlying secondary structures as cartoon. The distance between residues K176 and I342, shown in yellow as sticks, is plotted as a function of simulation time consecutively for all simulations on active PAI-1 in panel ***e)***. ***f)*** Backbone representation of β-strand 5A and β-strand 3A from the W175F crystal structure with hydrogen-bonds 169N:323O, 169O:325N (green), 171N:325O (blue), 171O:327N (red) and 173N:327O (black) indicated by dashed lines. ***g)*** hydrogen-bond lengths, according to the color coding in ***f)***, are plotted as a function of simulation time consecutively for all simulations on active PAI-1, as indicated.

Figure S9

a

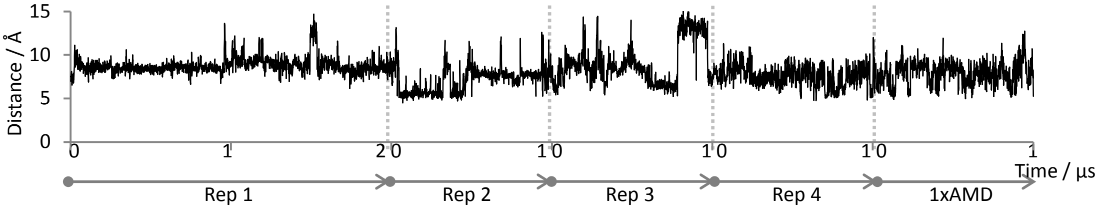

Figure S9: V157-loop dynamics in active PAI-1. The distances between C_α atoms of V157 and A318 plotted as a function of simulation time consecutively for all simulations on active PAI-1, as indicated**.**
